# Statistics of Grave-Yards

**Published:** 1855-09

**Authors:** John Webster


### STATISTICS OF GRAVE-YARDS,

### AS A GENERAL MEANS OF ASCERTAINING THE SALUBRITY OF PARTICULAR DISTRICTS.

By John Webster, M.D., F.R.S., &c.

Several methods have been adopted to ascertain the salubrity of different localities; the usual mode followed being to divide the aggregate amount of population by the total deaths annually recorded, and so establish a ratio, which may very fairly be reckoned as an average for that particular district. This system is no doubt excellent; but without detracting from such a procedure, my object in the present communication is to suggest another, or rather an additional manner of arriving at correct general conclusions on similar questions. The proposal mooted may not be new, and perhaps has been followed by other observers; nevertheless, having on variout occasions, when travelling through Great Britain, collected many facts of the kind, whereof it is proposed to give examples in subsequent pages, I am induced to bring this subject before the readers of the Journal of Health, and especially of its medical correspondents. The plan I suggest for ascertaining the health of towns and districts is, carefully to examine the gravestones in every churchyard of such localities; to note the ages of parties registered on these memorials of interments, and to report all particulars thus obtained in the JOURNAL OF HEALTH. Wherever numerous individuals are in this way stated to have attained an advanced period of life, having been, of course, also previously residents in the immediate neighbourhood, and especially wherever the proportion of such burials, compared with the cemetery's extent, seems large, then scarcely better evidence can be brought forward to prove the general healthiness of that particular locality, or the longevity of its inhabitants.

Considering it would be altogether superfluous to transcribe many observations recorded by myself, during numerous excursions I have made in England, to illustrate the question at issue: one or two extracts from these memoranda will suffice, in order to explain sufficiently my meaning; and should other, but more competent authorities forward for publication additional facts of the same description, such data would prove useful in detail, and become still more important when placed in juxta-position; since medical statists could in these cases arrive at highly instructive inferences, which it certainly would be very difficult, if not impossible, to deduce otherwise.

During a holiday tour which I enjoyed last autumn through several eastern counties, or speaking more historically, in East Anglia, the following rough notes were entered in my journal, respecting various church-yards inspected purposely. In the burying ground at Chipping Ongar, Essex, a number of persons there interred were 45 and 50, or even under that age, whilst very few ranged higher; consequently, this locality seemed of less than average salubrity. Cambridge appeared likewise high in the scale of mortality; many persons buried in this city being from 40 to 50, although 60 to 65 were also general; still, the grave-stones noticed rarely indicated that any exceedingly old persons reposed in the cemeteries of this far-famed classic soil, and ancient seat of learning.

In the episcopal church-yard of Ely, the ages mentioned were generally much higher than at Cambridge, 70 to 75 being frequent, from 80 to 85 by no means uncommon; and one female there interred had arrived at the great age of 102 years. This city hence occupies an elevated position in the longevity table, if viewed comparatively. Norwich likewise stands high; 75 to 80 and 85 being often observed on grave-stones, although numerous individuals much younger were similarly reported; upon which authority, it would seem, this populous city has hitherto contained not many very old people. Cromer, on the northern coast of Norfolk, holds a medium place as to longevity; the ages upon church-yard grave-stones, were sometimes from 70 to 75, but numbers were younger, while few reached beyond the first-named period.

Great Yarmouth, renowed for its bloaters, and at present much frequented by visitors during the summer weather, as a favourite sea-bathing quarter, in my estimation, appeared the most salubrious district, according to sepulchral data, which I examined in this county. In the extensive cemetery of that borough, 85 and 90

were common figures observed on the death memorials it contained, some of the persons buried being even above that advanced age, whilst one female, it was stated, had actually lived till her 111th year. These recorded facts consequently speak strongly in favour of the healthiness of this sea-port town.

Omitting several localities, I would now transcribe, but at rather greater length, the memoranda written at the time, regarding one more place likewise inspected last September, namely, Southwold, in Suffolk; about which, it is hoped, a special notice will not altogether be deemed uninteresting. This sea-port, known in history for the sanguinary conflict between an English and Dutch fleet in 1672 opposite its shores, and denominated the battle of "Sole Bay", besides being an agreeable marine residence, although situated on a small island partly made by the river Blything, must be salubrious, since, by its church-yard announcements, longevity, notwithstanding the hazardous life of fishermen, seems an ordinary lot amongst the inhabitants. On numerous grave-stones in its burying-ground, the persons interred were said to have attained their 70th year; many reached 80, or above that age up to 85 and 88; besides these, several had died at 90; and lastly, two were reported, according to the same evidence, as 93 years old at death. In addition to the above unmistakeable lithic memorabilia, it may be also added that, recently, a veteran fisherman died in this town upwards of 100 years of age, whose portrait I saw in the house of a grandson. This patriarch retained all his faculties to the last days of existence, and at 90, he often walked several miles consecutively. My informant further stated, in reference to this old man, that he remembered his own christening. This seems an odd circumstance, but it is easily explained. Having gone on foot to the church where that ceremony was performed, the fact became impressed on his memory; whilst the baptism being entered in the parish registers, this centenarian's exact age was accurately ascertained.

After alluding so briefly to the county of Suffolk, although it may seem rather digressing from the point more immediately under review, I would beg permission to advert to an interesting feature noticed, viz., that this division of England, besides being celebrated for an excellent breed of horses, is likewise remarkable on account of the tall stature and superior physical powers of conformation often observed in its native population. Without exaggeration, I have seldom seen more perfect outward specimens of the "genus homo" than persons of both sexes dwelling in Southwold and Saxmundham: the former a marine, the latter an inland situation. This peculiarity, common in Suffolk, must have frequently struck the eye of other tourists; and as analogous remarks may be justly applicable to various districts of East Anglia, strangers can readily understand the correctness of Pope Gregory the Great's well known observation respecting the ancient "Angles", when he first saw them exposed in the market-place of Rome.

Prior to concluding my cursory sketch of grave-stones, I would observe that various country districts adjoining London also merit inspection, in reference to the question in hand. Indeed, many churchyards around this great metropolis tell some strange tales of mortality, which are not only curious, but illustrate the actual sanitary condition of the districts. Several rural retreats so situated cannot boast of extraordinary salubrity, according to existing cemetery evidences. I shall only now, however, speak of four suburban districts, whose healthiness may bear comparison with other places adjacent, more particularly in regard to the longevity of inhabitants.

At Streatham, in Surrey, the churchyard grave-stones showed that many persons buried were middle-aged, some being also 70, others 80 or 85; and one individual was stated to have died when 90 years old. At the period I visited this village, several years past, a local acquaintance, cognizant of all the facts, told me that one man was then living in the parish aged 93, also a female at 96, whilst another woman had even reached her 101st year, and still enjoyed good health. These authentic data, therefore, indicate Streatham to be salubrious: and that it may be fairly quoted as somewhat pre-eminent for the long life which several of its residents have attained.

Eltham, in Kent, appears likewise healthy, compared with neighbouring districts, seeing the grave-stones in the burying-ground demonstrate by their records that a number of old people both resided and died here 75 and 80 years old. Eighty-five is not an uncommon age, and one or two, if I am correct, had passed their 90th year. The above details of this ancient royal domain, which, in addition to its favourable position on moderately elevated ground, which is open, airy, and has a south-western exposure, besides being protected from northerly winds by Shooter's Hill, make Eltham truly one of the most desirable localities near London, and a salubrious place of residence.

Hampstead, popularly known as a delightfully situated suburban village, often selected by invalids in warm weather, and of which one part is called the "Vale of Health", comes next under notice. In the old churchyard of this favourite resort for Londoners, its grave-stones show that many individuals there interred had attained the age of 70 and also 80, whilst others were even older. The tomb-stone commemorating a family named "Dove", particularly deserves inspection: since it states that, notwithstanding the mother died at 45, and the father at 48, one daughter lived till she was 72, another to 82; a son died at 80, and a second son at 90; and as some portion of the memorial in question remained unoccupied by any inscription when last I sauntered through this cemetery, it appeared as likely that the space left blank was intended for other members of the same family then alive. This grave-stone further possesses considerable value, as it bears upon the important question of hereditary longevity: seeing the above statement constitutes a rather unusual example of short-lived parents having very longlived offspring, several of whom lived to near twice the actual age of their progenitors. This marked result assumes, consequently, greater significance from contradicting the common, and, according to ordinary experience, more correct, opinion frequently entertained by actuaries regarding the probable duration of human life.

Mortlake churchyard, in Surrey, is the fourth and last locality, to which I would beg, on the present occasion, to make special reference. This rural cemetery appears singular on account of the numerous patriarchal people buried within its precincts. The gravestones show 70 to 80 as common ages. Several persons interred were stated to have been 84, 86, 88, and also 89, when they died. On one monumental slab the deceased party was reported to have lived 90 years; on another 92; whilst the oldest similar record met with referred to a female who had attained 95 years at her death. These data, therefore, conclusively indicate that this district is unusually salubrious, although it lies low, and nearly on a level with the adjacent river Thames. In support of the above conclusion as to Mortlake being considered a healthy residence, I may further report that my friend Dr. Scott, of Stratton-street, who practised during a long time in this neighbourhood, mentioned in conversation having, some years ago, professionally attended six patients simultaneously, all then living in or near Mortlake, of whom the youngest was 88, and the eldest in her 97th year. Dr. Richardson, who also resided for five years in Mortlake, tells me that there is living in an almshouse at Mortlake at this time, an old lady, named Hadlow, who, though bordering upon 100 years, retains all her faculties and takes her daily walk. Another woman, a neighbour of Hadlow's, and a patient of Dr. Richardson's, died suddenly from hæmoptysis when close upon her 90th year, while many other instances of longevity are here resident. Supported by such evidence, this district need not fear contrast with any other locality adjoining the metropolis, either in reference to salubrity, or the marked longevity of its inhabitants.*

Trusting these desultory pencilings—illustrative of graveyard statistics—may induce other inquirers to communicate their respective senectutal researches from the public repertories of the dead, I should be happy to correct whatever inaccuracies may have inadvertently crept into this narrative, which is submitted especially to the consideration of the medical profession: and is rather intended to direct attention to the question it embraces, than to be considered as claiming a higher or more authoritative pretension.

---

* Before taking leave of the mortuary memorials of Mortlake, I would briefly allude to a monument in the churchyard, which has occasionally attracted the inquisitive wayfarer's observation. This stone bears date A.D. 1715, and was erected to the memory of a man named Dr. John Partridge, said to be a graduate in medicine and an astrologer! but although he may have, perhaps, attained much popular repute during life, his former existence is now forgotten.

